# Laparoscopic and open resection for colorectal cancer: an evaluation of cellular immunity

**DOI:** 10.1186/1471-230X-10-127

**Published:** 2010-10-28

**Authors:** Chen Huang, Renxiang Huang, Tao Jiang, Kejian Huang, Jun Cao, Zhengjun Qiu

**Affiliations:** 1 Department of General Surgery, Affiliated First People's Hospital, Shanghai Jiao Tong University, Shanghai, PR China, 200080; 2 Digestive Endoscopy Center, Affiliated Huadong Hospital, Fudan University, Shanghai, PR China, 200040

## Abstract

**Background:**

Colorectal cancer is one kind of frequent malignant tumors of the digestive tract which gets high morbidity and mortality allover the world. Despite the promising clinical results recently, less information is available regarding the perioperative immunological effects of laparoscopic surgery when compared with the open surgery. This study aimed to compare the cellular immune responses of patients who underwent laparoscopic(LCR) and open resections(OCR) for colorectal cancer.

**Methods:**

Between Mar 2009 and Sep 2009, 35 patients with colorectal carcinoma underwent LCR by laparoscopic surgeon. These patients were compared with 33 cases underwent conventional OCR by colorectal surgeon. Clinical data about the patients were collected prospectively. Comparison of the operative details and postoperative outcomes between laparoscopic and open resection was performed. Peripheral venous blood samples from these 68 patients were taken prior to surgery as well as on postoperative days(POD) 1, 4 and 7. Cell counts of total white blood cells, neutrophils, lymphocyte subpopulations, natural killer(NK) cells as well as CRP were determined by blood counting instrument, flow cytometry and hematology analyzer.

**Results:**

There was no difference in the age, gender and tumor status between the two groups. The operating time was a little longer in the laparoscopic group (*P *> 0.05), but the blood loss was less (*P *= 0.039). Patients with laparoscopic resection had earlier return of bowel function and earlier resumption of diet as well as shorter median hospital stay (*P *< 0.001). Compared with OCR group, cell numbers of total lymphocytes, CD4^+^T cells and CD8^+^T cells were significant more in LCR group (*P *< 0.05) on POD 4, while there was no difference in the CD45RO^+^T or NK cell numbers between the two groups. Cellular immune responds were similar between the two groups on POD1 and POD7.

**Conclusions:**

Laparoscopic colorectal resection gets less surgery stress and short-term advantages compared with open resection. Cellular immune respond appears to be less affected by laparoscopic colorectal resection when compared with open resection.

## Background

Colorectal cancer is one kind of frequent malignant tumors of the digestive tract which gets high morbidity and mortality allover the world. Along with peoples' transition of life styles and food habits, the incidence rate of colorectal cancer gets rising for the past few years. Nowadays, surgery is still to remain as a principal method to treat colorectal cancer. Since the first laparoscopic cholecystectomy performed in France by Phillippe Mouriat of Lyons in 1987[1], the development of minimally invasive surgery has allowed major changes in the surgical treatment of various benign and malignant diseases, especially because it limits surgical trauma[2]. During recent years, the laparoscopic approach has developed as an interesting therapeutic alternative for the resection of various colorectal diseases[3-6]. In China, The first series of laparoscopic colorectal resection was reported by Shanghai Ruijin Hospital in 1993. Since 2000, there has been a remarkable increase in this field. Laparoscopic colorectal surgery has been routinely performed in most of the general teaching hospitals[7]. This procedure has been shown to be feasible in most patients with malignant disease and can be performed with faster recovery and shorter hospitalization than the open approach[8-11].

Despite the promising clinical results, less information is available regarding the perioperative immunological effects of laparoscopic surgery when compared with the open surgery. This prospective clinical study was of major clinical interest because the reduced surgical trauma should result in reduced postoperative immune, especially cellular immune dysfunction in patients undergoing laparoscopic surgery, thus contributing to clinical and oncologic advantages for these patients.

Until now, it has been reported that the degree of postoperative inflammatory is reduced after laparoscopic surgery[12,13]. Other groups also observed significantly better preservation of cell-mediated immunity after laparoscopic vs open colorectal surgery[14,15]. Furthermore, it has been observed that cell-mediated immunity, as assessed by delayed-type hypersensitivity testing in humans, is better preserved after laparoscopic vs open colorectal resection[16].

Our study was performed to evalute perioperative immune parameters in 68 patients with colorectal cancer. We herein analyzed the effects of laparoscopic and open surgery on proinflammatory C-reative protein(CRP) levels. Furthermore, we measured lymphocyte subpopulations, leukocyte and neutrophils counts, and circulating natural killer(NK) cells before surgery and on days1, 4, and 7 after surgery. This study, therefore, allowed assessment of the effects of laparoscopic and conventional open colorectal surgery on cellular immune responses after major abdominal surgery.

## Methods

### Patients

Between Mar 2009 and Sep 2009, 68 patients diagnosed with primary colorectal cancer were enrolled into this study. All patients, who underwent surgical treatment at Affiliated First People's Hospital of Shanghai Jiao Tong University, gave informed consent for the use of blood samples in this study. Among these prospectively enrolled patients, 35 underwent laparoscopic colorectal resection(LCR) and 33 underwent open colorectal resection(OCR).

Patients with colorectal cancer in stage IV intestinal obstruction, recurrence tumor, preoperative chemoradiation, palliative surgery and perioperative complications were excluded from the study. Patients with diabetes mellitus, use of steroids and with other immunological diseases were excluded from the study as well, since these events could impair the cellular immune responses after surgery.

Informed consent was obtained from all patients, who underwent surgical treatment at Affiliated First People's Hospital of Shanghai Jiao Tong University. Our research have been performed with the approval of the ethics committee of Affiliated First People's Hospital of Shanghai Jiao Tong University.

### Surgery Method

Minimally invasive colorectal surgery was performed as a laparoscopic-assisted procedure with removal of the resected specimen via a horizontal minilaparotomy (5 cm) just above the mons pubis. Laparoscopic surgery was done using a 5-trocar technique with 1 trocar (10 mm) inserted via a paraumbilical incision (camera port). Four additional (5 mm) trocars were inserted in the right and left lower abdomen. After removal of the resected specimen and preparation of the stapler anastomosis, we closed the minilaparotomy and reintroduced pneumoperitoneum.

Conventional colorectal surgery was performed via a vertical midline incision or a transrectal incision ranging from 10 to 15 cm above the umbilicus to the mons pubis. After we removed the resected specimen, we performed a stapler or a handwork anastomosis.

### Blood test

Peripheral venous blood samples were taken prior to surgery as well as on postoperative days(POD) 1, 4 and 7. Leukocyte number, absolute number and percentage of total lymphocytes for all the investigated patients were measured using an automated blood cell counter (Beckman LH750, America). CRP was measured using a CRP hematology analyzer (ABX Diagnostics Micros, France). The parameters of cellular immunity (CD3^+^, CD4^+^, CD8^+^, CD16^+^, CD56^+ ^and CD45RO^+^) were determined by flow cytometry (Beckman Coulter EpicsXL, America). The monoclonal antibodies used for immunophentyping were purchased from Beckman Coulter (France). The samples were prepared by labeling 50 μL of whole blood with 10 μL of monoclonal antibody for 10 minutes in the dark using the antibody combinations. Helper T lymphocytes were determined by UCHT1/13B8.2 indexed for CD3/CD4. Cytotoxic T lymphocytes were determined by UCHT1/B9.11 indexed for CD3/CD8. Natural killer cells were determined by 3G8/N901 indexed for CD16/CD56. Memory T lymphocytes were determined by UCHL1 indexed for CD45RO.

### Statistical analysis

Data were analyzed using SAS9.13 statistical package, analysis of variance on *t *test, Wilcoxon rank test and *χ*^2 ^test. Differences were considerd statistically significant at *P *< 0.05.

## Results

### Baseline characteristics

68 patients were enrolled into this study. Among the 35 LCR patients, 20 were male and 15 were female. The mean patient age was 68.43 ± 11.16 years in LCR group. Among the 33 OCR patients, 20 were male and 13 were female. The mean patient age was 68.33 ± 12.69 years in OCR group. There was no difference in the age, gender and tumor status between the two groups (Table 1).

**Table 1:** Baseline characteristics

	LCR (n = 35)	OCR (n = 33)	statistic	*P *value
Gender				
Male	20	20	*χ^2 ^*= 0.084	0.772
Female	15	13		
Age(yr)	68.43 ± 11.16	68.33 ± 12.69	*Z *= 0.055	0.956
Height(cm)	165.77 ± 8.23	162.36 ± 7.67	*t *= 1.760	0.082
Weight(kg)	63.30 ± 10.82	60.98 ± 11.33	*Z *= -1.032	0.302
BMI(kg/m^2^)	22.95 ± 2.88	22.91 ± 3.22	*t *= 0.060	0.956
Tumor position				
Left hemicolon	5	2	*χ^2 ^*= 0.002	0.564
Transverse colon	1	2		
Right hemicolon	9	13		
Sigmoid colon	7	4		
Rectum	13	12		
Tumor staging of TNM				
0	2	0	*χ^2 ^*= 0.009	0.405
I	2	3		
II	20	15		
III	11	15		
ASA classification				
I	2	0	*χ^2 ^*= -0.083	0.934
II	26	28		
III	7	5		

The baseline characteristics were similar between the two groups (*P *> 0.05).

### Perioperative complications

Between Mar 2009 and Sep 2009, in our department, 3 patients in LCR group and 2 patients in OCR group suffered from postoperative complications. In LCR group, anastomotic leakage occurred in 2 patients with low-set rectal cancer and paralytic ileus occurred in 1 patient with rectal cancer. All these 3 patients were cured after expectant treatment. In OCR group, incision disruption occurred in 1 patient with transverse colonic cancer and the patient accepted emergent suturation. The other patient with rectal cancer sufferring from paralytic ileus was cured after expectant treatment. There was no difference in postoperative morbidity between the two groups. Considering cellular immunity impaired by postoperative complications, which might impact cellular immune outcomes caused by selection of surgery, we excluded these 5 patients and selected 68 donors into this study at last.

### Surgery effects and postoperative recovery

The operating time was a little longer in the laparoscopic group (*P *> 0.05), but the blood loss was less (*P *= 0.039). Patients with laparoscopic resection had earlier return of bowel function and earlier resumption of diet as well as shorter median hospital stay (*P *< 0.001) (Table 2).

**Table 2:** Surgery effects and postoperative recovery

	LCR (n = 35)	OCR (n = 33)	*Z *value	*P *value
Operating time (min)	137.29 ± 34.58	130.61 ± 36.72	-0.752	0.452
Blood loss (ml)	117.27 ± 65.21	159.39 ± 83.40	-2.064	0.039*
Enterokinesia (d)	1.86 ± 1.09	3.00 ± 0.92	4.481	< 0.001*
Outgas (d)	2.54 ± 1.38	3.78 ± 1.18	4.144	< 0.001*
Out-of-bed activity (d)	2.80 ± 1.26	3.38 ± 1.07	2.385	0.017*
Fluid (d)	3.14 ± 1.35	5.27 ± 1.28	5.624	< 0.001*
Semifluid (d)	5.47 ± 1.50	7.97 ± 1.31	5.559	< 0.001*
Postoperative hospital stay (d)	9.88 ± 4.02	14.06 ± 1.46	5.896	< 0.001*
Total hospital stay (d)	17.91 ± 5.71	20.74 ± 2.42	3.740	< 0.001*

LCR group had more operating time (*P *> 0.05) but less blood loss (*P *< 0.05) than OCR group. Patients with laparoscopic resection had earlier return of bowel function and earlier resumption of diet as well as shorter median hospital stay (*P *< 0.05).

### Proinflammatory mediators

**Figure 1:**
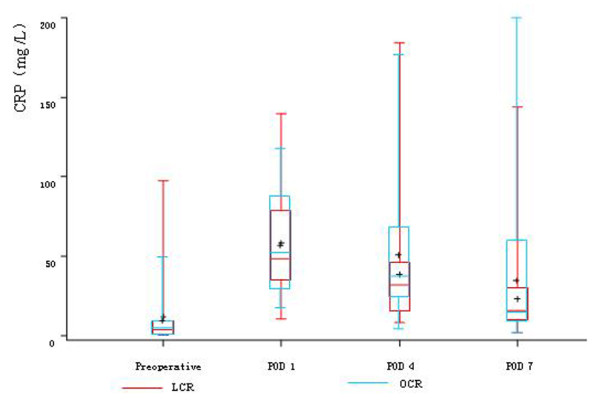
**Summary of CRP between the two groups**. On POD 1 and POD 4, the CRP levels were both significantly higher than the preoperative levels in both two groups (*P *< 0.05). But on POD 7, CRP descended to the preoperative level in LCR group (*P *> 0.05), while CRP was still significantly higher than the preoperative level in OCR group (*P *< 0.05).

After both laparoscopic and open colorectal surgery, we observed a significant increase of circulating CRP levels and this increase was similar between the two groups on POD 1 and POD 4. But on POD 7, CRP descended to the preoperative level in LCR group, while CRP was still significantly higher than the preoperative level in OCR group (Table 3, Figure 1).

**Table 3:** Summary of proinflammatory responses and cellular immunity

Immune mediators	LCR (n = 35)				OCR (n = 33)			
		
	Preoperative	POD 1	POD 4	POD 7	Preoperatvie	POD 1	POD 4	POD 7
WBC (× 10^9^/L)	6.10 ± 1.68	10.88 ± 2.29^#^	7.60 ± 1.93^#^	7.28 ± 1.46^#^	6.07 ± 1.45	12.21 ± 4.10^#^	8.24 ± 2.52^#^	7.96 ± 2.34^#^
Neutrophils (× 10^9^/L)	3.81 ± 1.62	9.24 ± 2.33^#^	5.58 ± 1.79^#^	5.06 ± 1.13^#^	3.89 ± 1.18	10.44 ± 3.91^#^	6.28 ± 2.19^#^	5.58 ± 1.81^#^
Lymphocytes (× 10^9^/L)	1.67 ± 0.52	0.99 ± 0.41^#^	1.24 ± 0.39^#^*	1.48 ± 0.43^#^	1.70 ± 0.56	1.16 ± 0.38^#^	1.06 ± 0.41^#^*	1.50 ± 0.54^#^
CD4^+^T cells (× 10^9^/L)	0.62 ± 0.25	0.32 ± 0.13^#^	0.51 ± 0.20^#^*	0.60 ± 0.23	0.62 ± 0.28	0.37 ± 0.20^#^	0.39 ± 0.21^#^*	0.59 ± 0.26
CD8^+^T cells (× 10^9^/L)	0.47 ± 0.24	0.28 ± 0.13^#^	0.33 ± 0.13^#^*	0.41 ± 0.16	0.43 ± 0.19	0.27 ± 0.13^#^	0.26 ± 0.13^#^*	0.38 ± 0.22^#^
CD45RO^+^T cells (× 10^9^/L)	0.52 ± 0.20	0.32 ± 0.14^#^	0.45 ± 0.15^#^	0.51 ± 0.17	0.55 ± 0.27	0.32 ± 0.17^#^	0.37 ± 0.18^#^	0.51 ± 0.27
NK cells (× 10^9^/L)	0.29 ± 0.21	0.16 ± 0.11^#^	0.17 ± 0.10^#^	0.23 ± 0.13^#^	0.32 ± 0.22	0.18 ± 0.16^#^	0.14 ± 0.14^#^	0.25 ± 0.22^#^
CRP(mg/L)	11.93 ± 20.56	58.38 ± 32.51^#^	38.43 ± 34.12^#^	23.18 ± 25.51	8.87 ± 12.44	53.77 ± 31.16^#^	48.83 ± 35.78^#^	27.19 ± 25.25^#^

^# ^*P *< 0.05, compared with preoperative levels;

* *P *< 0.05, compared between the two groups.

Especially, on POD 4, the count of lymphocytes, CD4^+^T cells and CD8^+^T cells were significantly higher in LCR group than OCR group (*P *< 0.05). The count of CD45RO^+^T cells in LCR group also had significant rise trend compared with OCR group (*P *> 0.05), while the count of NK cells in OCR group still had continuous depression trend compared with LCR group (*P *> 0.05).

### Markers of cellular immune responses

**Figure 2:**
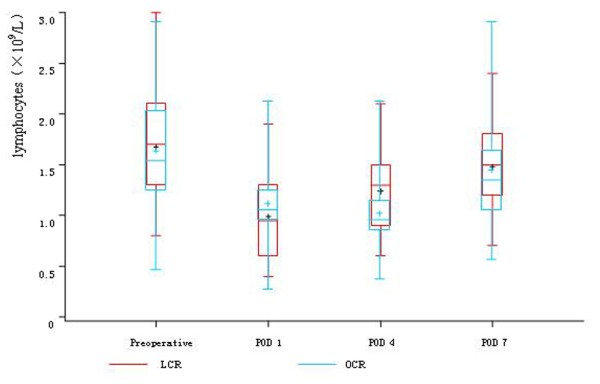
**Cell count of lymphocytes between the two groups**. On POD 1 and POD 7, in both two groups, the count of lymphocytes significantly descended (*P *< 0.05), while there was no difference between the two groups (*P *> 0.05). While on POD 4, the count of lymphocytes was significantly higher in LCR group than OCR group (*P *< 0.05).

**Figure 3:**
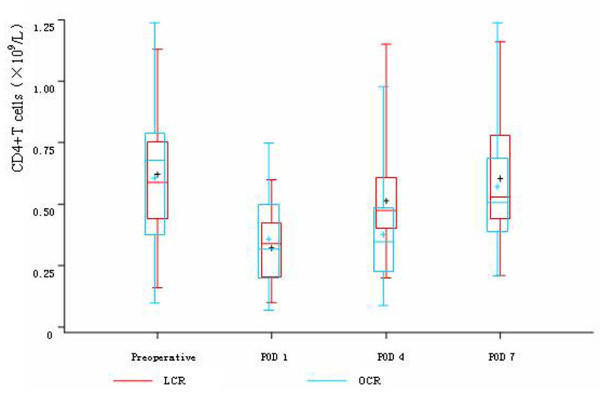
**Cell count of CD4^+^T cells between the two groups**. On POD 1, in both two groups, the count of CD4^+^T cells significantly descended (*P *< 0.05), while there was no difference between the two groups (*P *> 0.05). While on POD 4, the count of CD4^+^T cells was significantly higher in LCR group than OCR group (*P *< 0.05). On POD 7, the count of CD4^+^T cells rose to the preoperative level in both two groups.

**Figure 4:**
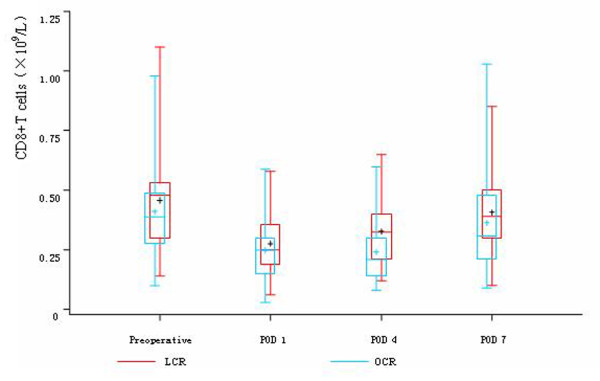
**Cell count of CD8^+^T cells between the two groups**. On POD 1, in both two groups, the count of CD8^+^T cells significantly descended (*P *< 0.05), while there was no difference between the two groups (*P *> 0.05). While on POD 4, the count of CD8^+^T cells was significantly higher in LCR group than OCR group (*P *< 0.05). On POD 7, the count of CD8^+^T cells rose to the preoperative level in LCR group, while in OCR group, the count of CD8^+^T cells was still significantly depressed compared with the preoperative level (*P *< 0.05).

**Figure 5:**
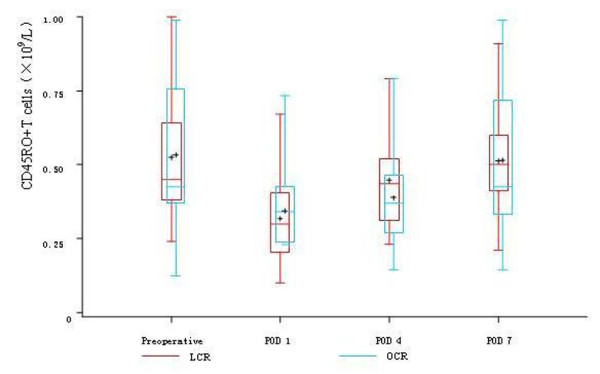
**Cell count of CD45RO^+^T cells between the two groups**. On POD 1, in both two groups, the count of CD45RO^+^T cells significantly descended (*P *< 0.05), while there was no difference between the two groups (*P *> 0.05). While on POD 4, The count of CD45RO^+^T cells in LCR group had significant rise trend compared with OCR group (*P *> 0.05). On POD 7, the count of CD45RO^+^T cells rose to the preoperative level in both two groups.

**Figure 6:**
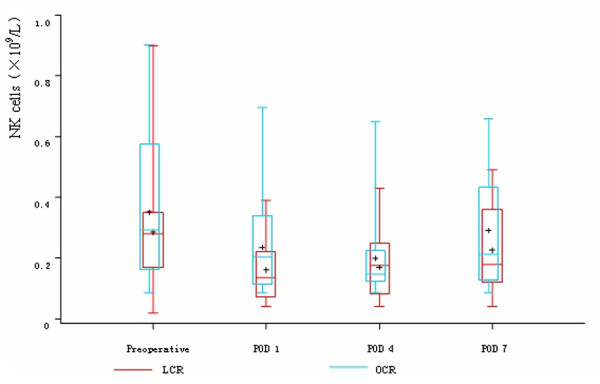
**Cell count of NK cells between the two groups**. On POD 1, in both two groups, the count of NK cells significantly descended (*P *< 0.05). While on POD 4, the count of NK cells in OCR group still had continuous depression trend compared with LCR group (*P *> 0.05). On POD 7, the count of NK cells still less than the preoperative level in both two groups (*P *< 0.05), but there was no difference between the two groups.

Before the operations, there was no difference in the count of leukocyte, neutrophils, lymphocytes, CD4^+^T cells, CD8^+^T cells, CD45RO^+^T cells and NK cells between the two groups. On POD 1, in both two groups, the count of leukocyte and neutrophils significantly rose and the count of lymphocytes, CD4^+^T cells, CD8^+^T cells, CD45RO^+^T cells and NK cells significantly descended, while there was no difference between the two groups. On POD 4, the count of lymphocytes, CD4^+^T cells and CD8^+^T cells were significantly higher in LCR group than OCR group. The count of CD45RO^+^T cells in LCR group also had significant rise trend compared with OCR group, while the count of NK cells in OCR group still had continuous depression trend compared with LCR group. On POD 7, the count of CD4^+^T cells, CD8^+^T cells and CD45RO^+^T cells rose to the preoperative levels in LCR group. In OCR group, the count of CD4^+^T cells and CD45RO^+^T cells also rose to the preoperative levels, while the count of CD8^+^T cells was still significantly depressed compared with the preoperative level (Table 3, Figure 2, 3, 4, 5 and 6).

## Discussion

Colorectal cancer is one kind of frequent malignant tumors of the digestive tract which gets high morbidity and mortality all over the world. Nowadays, surgery is still to remain as a principal method to treat colorectal cancer. On the other hand, tumor immunity, mainly defined as the immune responses in the body against tumor, acts an important role during the process when patients fight against tomors. When tumor antigens were recognised by antigen presenting cells(APC), they were processed as peptides, and these peptides presented by APC acted as first and second signals to activate naive CD4^+^T cells and CD8^+^T cells. As naive CD4^+^T cells and CD8^+^T cells received these signals, they differentiated into Th0 cells and pCTL. When Th0 cells received different allostimulatory signals, they were activated as Th1 and Th2 cells. IL-4 secreted by Th2 cells acted as allostimulatory signal to activate B cells. Activated B cells could secrete generous tumor specific antibodies and killed tumor cells through complement dependent cytotoxicity(CDC) and antibody dependent cell-mediated cytotoxicity(ADCC). IFN-γ secreted by Th1 cells acted as allostimulatory signal to activate pCTL. Activated CTL could kill tumor cells directly. Besides, IFN-γ secreted by Th1 cells could enhance the activity of NK cells and Mφ, which could fight against tumors through ADCC. After antitumor immune responses, part of activated T cells could differentiate into memory T cells(CD45RO^+^T cells), which had quick reaction against tumor cells in twice antitumor immune responses[17,18]. It can be seen that cellular immunity acts an predominant role in tumor immunity. Although surgery is regarded as the most important way in tumor therapy, many reports have confirmed that surgery stress has great depressant effect on cellular immunity in the body, which would bring negative influence to prognosis of patients[16,19,20]. So to think of a way to decrease surgery stress as well as the influence of cellular immunity becomes quite important.

The development of minimally invasive surgery has allowed major changes in the surgical treatment of various benign and malignant diseases, especially because it limits surgical trauma. Since Jacobs[21] reported the first case of laparoscopic sigmoidectomy, during recent years, the laparoscopic approach has developed as an interesting therapeutic alternative for the resection of colorectal cancer. Because the surgical trauma is limited, the laparoscopic approach usually allows for a rapid return to preoperative activity levels with significantly shorter hospitalization.

This study found that the operating time of LCR group and OCR group were 137.29 ± 34.58 min and 130.61 ± 36.72 min, prospectively. There was no difference between the two groups. Conventional versus laparoscopic assisted surgery in colorectal cancer (CLASSICC)[22], Colon cancer laparoscopic or open resection study group (COLOR)[6] and Lezoche[8] also observed similar results. Our findings shew that the intraoperative blood loss in LCR group and OCR group were 117.27 ± 65.21 ml and 159.39 ± 83.40 ml, prospectively. We found the intraoperative blood loss was much less in LCR group. Barcelona[5] and COLOR[6] also observed similar outcomes as we did. Patients with laparoscopic resection had earlier return of bowel function and earlier resumption of diet as well as shorter median hospital stay. This observation confirmed findings reported by Breukink[23]. Our findings suggested that short-term quality-of-life benefits could be observed with LCR when compared with OCR.

Besides the promising clinical results, we focused on the perioperative cellular immunological effects of LCR when compared with OCR.

Many researches have confirmed that prognosis of cancers depends on invasion of tumors and immunoreaction of bodies. Paholyuk[24] found that NK cells played an important role in suppressing growth of colorectal cancers of stage II. Milašienė[25] reported that counts of CD4^+^T cells, CD8^+^T cells and NK cells directly correlated with long-term overall survivals of patients with colorectal cancers and gastric cancers of stage III.

Hiki[12] reported that in laparoscopy there was generally less manipulation and exposure of the intestine than in open surgery, with a further reduction in host inflammatory responses. Our findings shew that minimally invasive surgery resulted in a less pronounced proinflammatory response to surgical trauma. Jung[13] also got similar results in the research, who found laparoscopic surgery got less influence on CRP than open surgery.

Many researches have observed that there is a distinct immunologic advantage to laparoscopic surgery[26-29]. Our findings confirmed better preserved cellular immune responses in patients undergoing laparoscopic colorectal resections. The counts of CD4^+^T cells, CD8^+^T cells, CD45RO^+^T cells and NK cells were more in LCR group than OCR group, especially on POD 4. This observation was in agreement with the findings of Whelan[16], who also observed better preserved cellular immune responses in patients undergoing laparoscopic surgeries. Although our findings were not completely comparable with the researches from Wichmann[14], we found similar change tendency of NK cells. Han[15] also reported that laparoscopic resections got less influence on cellular immune functions of the patients with colorectal cancers of stage III. Peng[30] found the perioperative immune response was less obvious after a laparoscopic procedure compared with a conventional approach in patients with renal cell carcinoma.

Compared with the previous research articles, our study more comprehensively reflected the progress of laparoscopic colorectal cancer radical resections and the improvement of cellular immune protection nowadays in Mainland of China.

There is a suggestion that cancers demonstrate a more aggressive phenotype after open surgery, with the more profound immunosuppression contributing to more rapid cancer growth[31]. Milašienė[25] reported that better cellular immunity correlated with higher postoperative survival rates in patients. The long-term effects of better preserved cellular immune responses and its relationship with the prognosis of patients still remain obscure. However, we can assume that our observation of better preserved cellular immunity in patients after laparoscopic colorectal surgeries has beneficial effects on the prognosis of patients.

## Conclusions

In conclusion, our study demonstrates that laparoscopic colorectal resection gets less surgery stress and short-term advantages compared with open resection. Also, cellular immune respond appears to be less affected by laparoscopic colorectal resection when compared with open resection.

## Competing interests

The authors declare that they have no competing interests.

## Authors' contributions

QZJ supervised the design of the experiments and carried out all the operations. HC conceived the study and drafted the manuscript. HRX was involved in blood test, drafting of the manuscript and design of the study. JT analysed and interpreted of data. HKJ and CJ participated in the design and coordination of the work involved. All authors read and approved the final manuscript.

## Pre-publication history

The pre-publication history for this paper can be accessed here:

http://www.biomedcentral.com/1471-230X/10/127/prepub
